# Hybrid Eye-Tracking on a Smartphone with CNN Feature Extraction and an Infrared 3D Model

**DOI:** 10.3390/s20020543

**Published:** 2020-01-19

**Authors:** Braiden Brousseau, Jonathan Rose, Moshe Eizenman

**Affiliations:** 1 Department of Electrical and Computer Engineering, University of Toronto, Toronto, ON M5S 1A4, Canada; Jonathan.Rose@ece.utoronto.ca (J.R.); eizenm@ecf.utoronto.ca (M.E.); 2 Ophthalmology and Vision Sciences, University of Toronto, Toronto, ON M5T 3A9, Canada; 3 Institute of Biomaterials and Biomedical Engineering, University of Toronto, Toronto, ON M5S 3G9, Canada

**Keywords:** eye-tracking, gaze estimation, smartphone, convolutional neural network, machine learning

## Abstract

This paper describes a low-cost, robust, and accurate remote eye-tracking system that uses an industrial prototype smartphone with integrated infrared illumination and camera. Numerous studies have demonstrated the beneficial use of eye-tracking in domains such as neurological and neuropsychiatric testing, advertising evaluation, pilot training, and automotive safety. Remote eye-tracking on a smartphone could enable the significant growth in the deployment of applications in these domains. Our system uses a 3D gaze-estimation model that enables accurate point-of-gaze (PoG) estimation with free head and device motion. To accurately determine the input eye features (pupil center and corneal reflections), the system uses Convolutional Neural Networks (CNNs) together with a novel center-of-mass output layer. The use of CNNs improves the system’s robustness to the significant variability in the appearance of eye-images found in handheld eye trackers. The system was tested with 8 subjects with the device free to move in their hands and produced a gaze bias of 0.72°. Our hybrid approach that uses artificial illumination, a 3D gaze-estimation model, and a CNN feature extractor achieved an accuracy that is significantly (400%) better than current eye-tracking systems on smartphones that use natural illumination and machine-learning techniques to estimate the PoG.

## 1. Introduction

The capability to estimate where a subject is looking is known as gaze estimation or eye-tracking. This technology has enhanced applications in a wide array of domains, including the measurement of advertising efficacy [1,2], instrumentation to enhance reading [3,4,5], automotive safety [6,7], pilot training [8,9,10], accessibility interfaces [11,12,13,14], and provide objective indicators of cognitive, psychiatric, and neurological states of individuals [15,16,17,18,19,20,21,22,23,24,25,26]. Even though research with specialized eye-tracking systems demonstrated that such systems can be used in many domains, the need to purchase expensive, specialized software and hardware to monitor the PoG limit the use of eye-tracking systems by consumers. In this paper, we describe an eye-tracking system that was integrated into a modern smartphone; the most widely accessible and deployed personal computing platform [27] in history. The paper describes a smartphone-based eye-tracking system that is accurate and relatively insensitive to motion between the device and the user’s head. Due to the increasing body of research regarding what can be learned about mental states of an individual though analysis of their gaze, high-quality eye-tracking becoming a pervasive sensor in most devices (just as GPS and accelerometers have) would present another layer of privacy concerns for users of these devices [28]. However, the potential large positive impact of greater access to eye-tracking warrants continuing research and development of the core technology.

**Figure 1:**
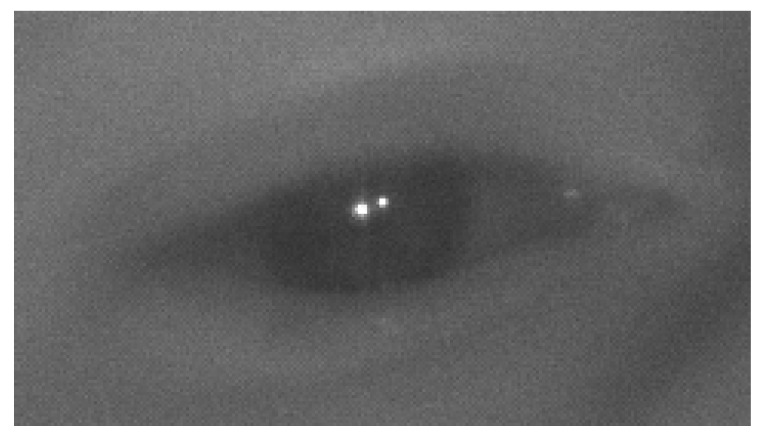
Infrared Eye Image with Corneal Reflections.

The most accurate video-based eye-tracking systems [29] use methods that rely on artificial illumination of the eyes with infrared (IR) light and the estimation of eye features (e.g., the center of the pupil) in images that are captured by IR sensitive cameras, as shown in Figure 1. These systems can measure the PoG with bias that is lower than 0.5° [29]. As IR illumination sources and IR sensitive cameras are not yet standard in commercial smartphones, all the prior work on smartphone-based eye-tracking system has used techniques that rely on visible light illumination of the eye (either by sun light or light emitted from the display) [30,31,32,33,34,35,36,37]. A history of gaze enabled handheld mobile devices which use visible light illumination of the eye can be found in [28]. Even under favorable operating conditions these systems have reported gaze bias that is larger than 3°. Smartphones may not always face the current limitations with respect to IR light sources and IR sensitive cameras, and indeed IR light sources and IR sensitive cameras have begun to appear in some commercial smartphones to enhance face tracking and authentication systems [38,39]. While the manufacturers of these smartphones do not yet make the IR components available to third party software developers, the devices are otherwise essentially ready to enable IR-illuminated-based eye-tracking. Apple, as part of its augmented reality framework (ARKit [40]) has now introduced calibration-free gaze estimation as an API for iPhoneX.

In this paper, we explore the performance and viability of accurate IR model-based eye-tracking in a smartphone the mobile form factor, which moves significantly relative to the user during use. We then compare those results to the best available mobile eye-tracking systems which are presented in the literature.

### 1.1. Smartphone-Based Eye-Tracking Systems That Use Visible Light

Several eye-tracking systems that are based on a smartphone platform and use visible light are described in the literature [30,31,32,33,34,35,36,37]. One of these systems, GazeCapture [35], uses natural light (e.g., room illumination) and images that are captured by the front-face camera of the smartphone to generate estimates of the PoG. The system uses a deep convolutional neural network (CNN) to process images of the subject’s face and the point-of-gaze estimate is produced by the CNN. In GazeCapture [35] The CNN was trained with tens of thousands of labeled images from nearly 1500 people. The large training set that spans the expected range of natural variations in head pose and room lighting at home or in the office makes the system robust to novel faces in these environments. The GazeCapture [35] eye-tracking system has a gaze-estimation bias that is greater than 3° [35].

Another, smartphone-based eye-tracking system, ScreenGlint [36], uses a corneal reflection that is created by the light emitted by the smartphone’s display to generate PoG estimates that are based on the distance between the iris center and this corneal reflection. For a stationary eye-tracking system (i.e., the smartphone is not moving) ScreenGlint [36] reported a gaze-estimation bias of 2.9°. As discussed in [29] an approach that using a single corneal reflection and the center of the pupil/iris is not inherently robust to head or device motion, so the accuracy of the system will deteriorates during typical use when the smartphone will be handheld and free to move.

### 1.2. Smartphone-Based Eye-Tracking Systems That Use Artificial Infrared Illumination

Gaze-estimation systems that require artificial infrared illumination and infrared cameras have demonstrated both high accuracy and robustness to motion [41]. In these systems the location of eye features (the center of the pupil and two or more corneal reflections) are used to determine gaze position. These systems use a 3D gaze-estimation model [29,42] which make the PoG estimates robust to relative motions between the head and the smartphone. In a previous study [43] we used this approach to achieve an average gaze-estimation bias for a handheld mobile eye-tracker on a smartphone of 1°. Closer examination of our results showed that even though the gaze-estimation method is insensitive to movements between the eye-tracker and the subject’s head, the bias in gaze estimations did change as a function of the distance between the head and the smartphone from between 0.4° to 2.1°. The main reason for these significant changes was the system’s inability to accurately estimate the positions of the pupil center and corneal reflections at the full range of the expected relative positions of the head and the smartphone during regular use. Dramatic changes in the appearance of the face and the eyes during regular use make this task particularly challenging. In this paper, we address this issue by developing machine-learning algorithms (convolutional neural networks—CNNs) that can estimate the position of eye features accurately for the full range of expected smartphone motions. The approach is similar to that used for the detection and estimation of facial features [44,45,46,47]. The eye-feature extractor uses a hierarchical cascade of CNNs and a center-of-mass output layer to estimate the position of the eye features. With this structure, the locations of the corneal reflections and pupil center can be estimated with sub-pixel accuracy and negligible bias while the computation is efficient enough to run on a smartphone. As our approach involves both the use of machine-learning models and geometric gaze-estimation models, we refer to this as a ‘hybrid’ approach to gaze estimation.

## 2. Materials And Methods

### 2.1. Overview of the End-to-End Mobile Eye-Tracking System

**Figure 2:**
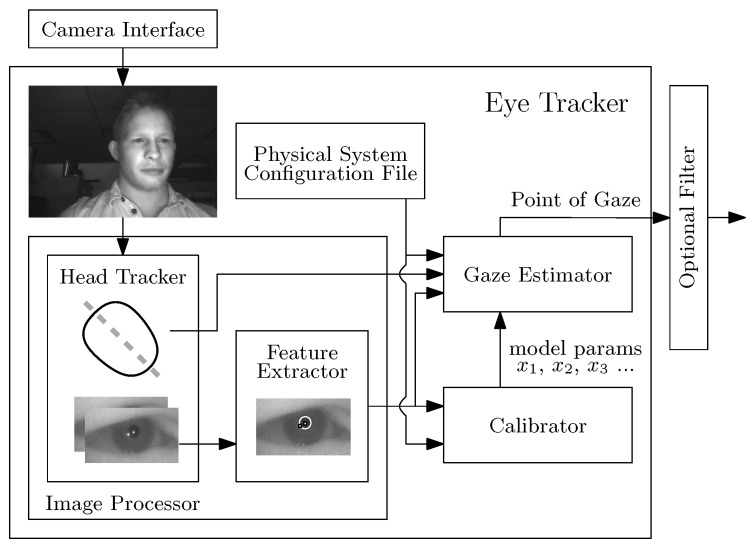
Eye-Tracking System [42], licensed under https://creativecommons.org/licenses/by/4.0/.

The top-level overview of our hybrid infrared smartphone-based eye-tracking system, reviewed here, is similar to the first-generation system, described in [43], and makes use of the gaze-estimation model presented in [29]. The four principal software components of the eye-tracker are a head tracker, a feature extractor, a calibrator, and a gaze estimator. Figure 2 illustrates the flow of data between the software modules. We run these software modules on an industrial infrared prototype smartphone, provided to us by Huawei [48].

#### 2.1.1. Infrared Smartphone

**Figure 3:**
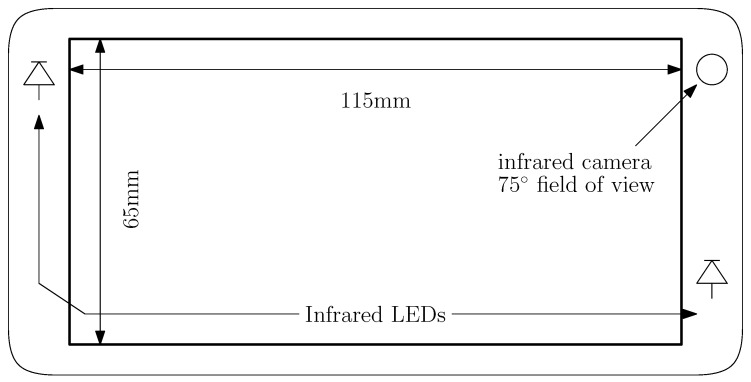
Prototype Infrared Device.

The smartphone includes two infrared LEDs that illuminate the user’s face together with and infrared-sensitive front-facing camera. Huawei [48] integrated these components into an industrial prototype smartphone explicitly to explore mobile eye-tracking in future products. The front-facing camera has a 4 K resolution, which is similar to high-end current Android devices. It also has a 5-inch display, 1.2 GHz processor, and 1 GB of RAM, which makes it both smaller and significantly less powerful than modern phones. Figure 3 illustrates the layout of our device.

#### 2.1.2. Head Tracker

A commercial head tracker [49] is used to identify the presence of a face and the approximate locations of the eye regions. These regions initialize the feature extractor from a cold start or when local tracking fails. For this reason, the system uses the head tracker only occasionally. When the eye features were tracked successfully in the previous frame, the regions that initialize the feature extractor are centered on the estimation of the previous center of the pupil. Localization of narrow eye region windows is done though a hierarchy of small efficient CNNs as discussed in Section 2.2.

#### 2.1.3. Calibrator

The calibration procedure determines four physical geometric properties of each user’s eyes as described in [29]. These parameters are; (1) the radius of curvature of the cornea, (2) the distance between the center of corneal curvature and the center of the pupil, (3) the vertical offset angle between the optical and visual axis, and (4) the horizontal offset angle between the optical and visual axis. To perform calibration, and to estimate the values of these four parameters, a user is required to gaze at two (or more) known target locations. Then, starting with physiologically average human values as a seed, the four parameters are varied with a nonconvex optimizer. Gaze estimates are generated for each target and the average Euclidean distance between the gaze estimates and their corresponding targets is minimized. Once a calibration procedure has been completed once for a subject, these parameters can be saved and reused for future uses of the eye-tracker by the same person.

#### 2.1.4. Gaze Estimator

The gaze estimator uses a 3D gaze-estimation model that takes in the user-specific eye geometry [29] and the locations of the pupil center and two corneal reflections in the eye-images to estimate the PoG. The gaze-estimation model is invariant to both device and head movements [29,42].

#### 2.1.5. Feature Extractor

The feature extractor estimates the locations of the pupil center and two corneal reflections in eye-images from the front-facing camera of the smartphone. Its design is presented in the next section as it is a key contribution of this work.

### 2.2. Feature Extractor Architecture

The feature extractor consists of a hierarchy of multiple independently trained CNNs. One of the CNNs operates as an eye region classifier, and the rest directly estimate feature locations. The classifier network determines if both corneal reflections are visible and that the pupil is not significantly occluded (such as when a user blinks). If this network determines that the eye region is valid, several independent position estimation networks determine the location of the pupil center and corneal reflections.

**Figure 4:**
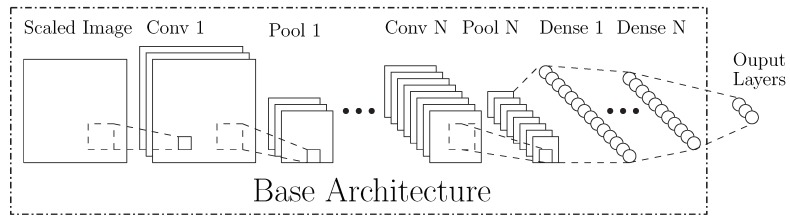
Base CNN Architecture.

The classifier and position estimation networks use similar base CNN architectures, as shown in Figure 4. They contain several convolutional layers (with batch normalization and optional pooling) followed by fully connected dense layers. Within this base architecture, there are many configurable hyperparameters (see Table 1) but the primary architectural differences between the networks explored in this work occur at the output layer, the loss function, and the encoding of ground truth. The subsequent discussion of both the classifier and position estimation networks will focus on these.

#### 2.2.1. Classifier

**Figure 5:**
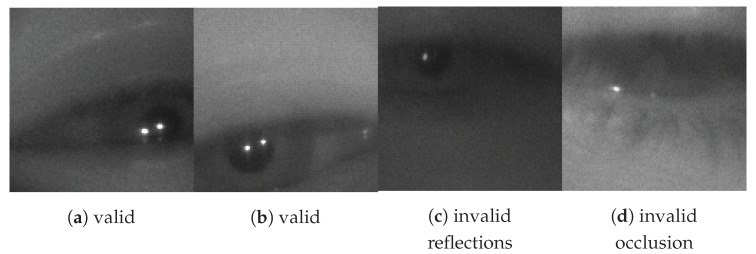
Example Valid (a-b) and Invalid (c-d) Eye Region Images From Dataset (128 × 128 pixels).

**Figure 6:**
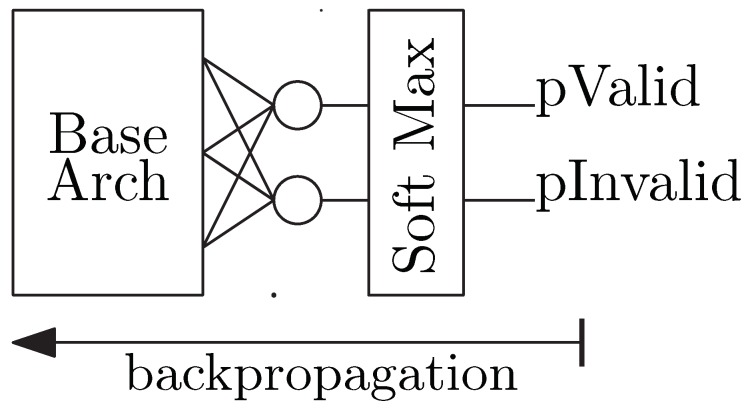
Eye Region Classifier Architecture.

The classifier is responsible for determining if an image of an eye region is valid (i.e., it contains two corneal reflections and a pupil). Figure 5 illustrates examples of valid and invalid eye region images. The output layer for the classifier, shown in Figure 6, extends the base architecture with a dense layer containing two neurons, one for each of the two classes: invalid or valid. The final layer of neurons is followed by a SoftMax function to generate the probability that the given image belongs to each class. The ground truth for each training sample is a one-hot encoded vector indicating the class of the example as either a valid eye region or an invalid region. The loss function is a cross-entropy function that sums the log of the difference between each predicted class probability and the ground truth. This approach is typical for image classification [50]. The cross-entropy equation for a binary classification is shown in Equation (1), where y is a binary indicator of valid/invalid eye region class label, p is the predicted probability of an eye region being valid, and N is the size of the training batch. No further processing happens for eye regions classified as invalid. Otherwise, the next step is to determine the locations of the pupil center and the two corneal reflections.

(1) $$\sum_{i=1}^{N}-\left(y_{i}*log\left(p_{i}\right)+\left(1-y_{i}\right)*log\left(1-p_{i}\right)\right)$$

#### 2.2.2. Feature Estimator

**Figure 7:**
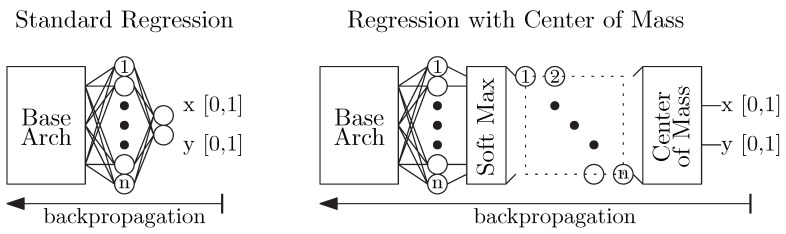
Eye-Feature Locator Networks.

The position estimation networks are crucial for achieving high accuracy and robust mobile eye-tracking. A standard approach to position estimation is the construction of regression type networks, as illustrated in Figure 7. This approach extends the base architecture with a final two-neuron dense layer representing a predicted (x,y) relative location of the feature in the input image. For example, an inference output of [0.5, 0.5] would indicate the feature was in the center of the input image. The ground truth for each training sample is the human-labeled relative (x,y) location of the feature. The objective when training the network is to minimize the sum of the Euclidean distances between the predicted locations and the ground truths for a given training batch. It is computed with Equation (2), where N is the number of eye regions in the training batch, $x_{i}$ and $y_{i}$ are the estimated x and y location for a particular sample and $gtx_{i}$ and $gty_{i}$ are the ground truth labeled x and y location for a sample. At a higher level, we care about selecting a network which optimizes the accuracy of the position determination while being constrained by the computational and memory footprint limitations imposed by the desire to operate in real time on a smartphone. Two factors that play an important role here are the design of the output layer and the choice of the input image size.

(2) $$\sum_{i=1}^{N}\sqrt{{\left(x_{i}-gtx_{i}\right)}^{2}+{\left(y_{i}-gty_{i}\right)}^{2}}$$

**Figure 8:**
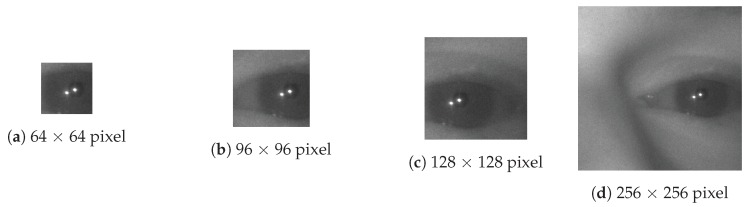
Example Eye Region With A Varying Native Crop Size (in pixels).

The input image size plays an essential role in attaining both high accuracy and computationally efficient position estimation networks. It is vital to avoid excessive down-sampling the input image to achieve the highest accuracy because of the reduction of precision that such down-sampling implies. A direct, unscaled, crop of the original image near the eye region should be used if possible. Figure 8 shows four examples of crop sizes that could be used for training a pupil center estimator. Crop size refers to the size of the window, which is taken from the original image, while down-scaling refers to how much that cropped image is reduced in size, through scaling, before sending it to the network. For example, if we compare a 256 × 256 crop down-scaled by four times with a native 64 × 64 crop that is not down-scaled (of the same eye region) both images have the same number of pixels. However, the size of the pupil in the native 64 × 64 crop will be four times bigger than the alternative. To maintain the same spatial resolution of the pupil we might choose not to downscale the 256 × 256 crop but now the network will be more expensive to run.

We explored four image crop sizes and determined that a smaller input image size (a tighter crop around the eye region) results in less overall computation and produces (as shown later in a Section 3) a *more accurate* estimate of the pupil center. The tighter crop requires that the system must already know the approximate pupil center. To solve this problem, we used a sequence of small CNNs to focus in on the rough pupil-center position successively. We train these networks on aggressively sub-sampled (scaled) images of larger regions around the eye. In these networks, we trade accuracy for speed/computational effort, because we only need to determine an area that contains the eye and not the precise location. Using this type of hierarchy, we estimate the pupil center to within a few pixels. Then, from a very tightly cropped region around that location, more computationally expensive and precise locator networks find the exact pupil center and corneal reflections. A key feature of which is the construction of the output layer.

The output layer of a regression network can also have a positive impact on performance, which is to say variations here can improve the position estimation accuracy while only negligibly increasing overall computation or memory footprint of the entire network (including the base architecture). In this work, the output layer is a dense layer with one neuron per pixel of the input image, followed by a SoftMax layer, and a two-dimensional center-of-mass computation (Figure 7). In this approach, we treat the position estimation problem as a classification problem by making a network which maps an output neuron to each input pixel. Each neuron outputs the probability that any specific pixel was the target location. During a forward inference pass, there are many pixels in the area around ground truth location that will have some significant non-zero probability of being the inferred feature location. Therefore, rather than merely selecting the pixel with the highest probability, we apply a two-dimensional center-of-mass calculation to that set of probabilities.

**Figure 9:**
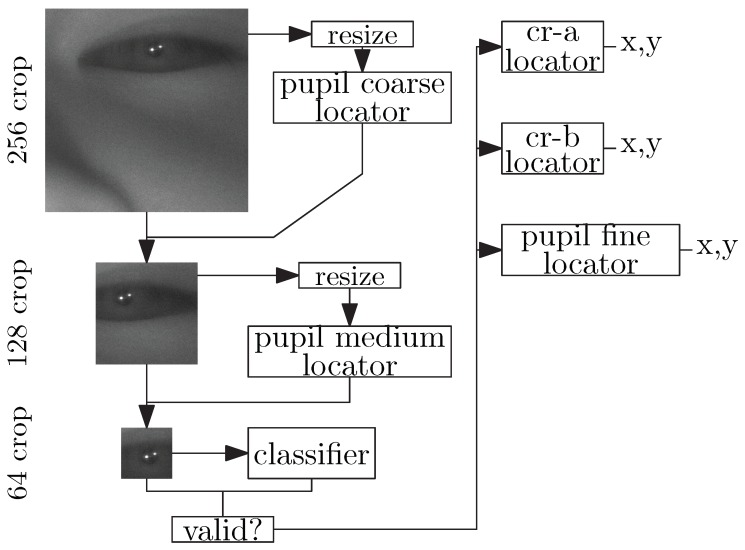
Multiple Network Feature Estimator Hierarchy.

In Section 3, we will show that our center-of-mass output architecture improves feature estimation accuracy to computational cost ratio relative to the naive regression approach. Figure 9 shows the complete architecture of our feature extractor hierarchy. It consists of six total networks: two very fast, but approximate, coarse pupil estimators which localize successively tighter eye regions. Next is a classifier to determine if the generated tight crop is a valid eye region. Finally, the classifier is followed by three accurate position estimators to determine the exact position of the pupil center and both corneal reflections. These positions are then given to the gaze-estimation model to compute the PoG.

The next section discusses how we trained, evaluated, and selected the specific feature extractor networks that we eventually integrated into our end-to-end eye-tracking system.

## 3. Feature Extractor Training And Performance

In this section, we outline the training, analysis, and selection of the feature extractor CNNs described previously, beginning with the collection of a dataset for training.

### 3.1. Dataset Collection

Our training dataset consists of infrared images of eye regions from the smartphone’s front-facing camera with labeled positions of the eye features. A dataset of this kind did not yet publicly exist, so we had to collect a new one. The image variability we expect in a smartphone environment comes mainly from the relative position and orientation changes between the device and the subject’s face. There can also be higher variability because of the (possibly) weaker contrast between the pupil and iris, which is caused by limited illumination power and a noisier camera sensor.

We collected and labeled a total of 2000 face images (4000 eye regions) from 100 participants without glasses (without controlling for contacts) holding our prototype infrared smartphone. Each participant was asked to position the device anywhere that they felt comfortable and look at a random target on the display. The device captured a single photo. Then the subject was given five seconds to re-position the device and repeat the same procedure before the next image was captured. The positioning and re-positioning of the device resulted in a variety of relative distance and orientations of the device and the participants’ heads. This process repeated 20 times for each subject, resulting in 20 infrared images of their face captured by the front-facing camera. During the 20-image collection process a random set of 6 frame indexes are generated for the purpose of artificially increasing the number of invalid eye regions in the dataset. When capturing these specific frames either one or both infrared LEDs (chosen at random) are disabled resulting in eye regions unsuitable for the gaze-estimation model used in this work. For the other 14 frames both LEDs were enabled. The orchestration of the LEDs in this fashion was done automatically by the sample collection app.

Re-positioning of the device resulted in a good variation of the relative distance and orientation between the device and the participants’ head. If the subject held the device in such a way that their eyes were not somewhere in the frame than they were informed of this by the experimenter In practice this occurred less than 10 times in the collection of 2000 samples. This is due, in part, to the wide field of view on the front-facing camera of our prototype smartphone of 75° combined with the active task of looking at a target. Data collection occurred exclusively in indoor environments with minimal natural light interference (although no specific controls were employed to avoid occasional direct sunlight from windows).

In the first stage of labeling each eye region is labeled with either a valid eye or an invalid eye class label. An eye region is considered ‘invalid’ if the pupil is significantly occluded, a corneal reflection is missing, or the image is exhibiting significant motion blur. Invalid eye regions can naturally occur during a blink or when images are captured during large device motion. Examples of eye regions labeled as valid and invalid are shown in Figure 5. The class distribution between valid and invalid eye regions in the 2000 captured images is approximately balanced, with 55% valid and 45% invalid. In many cases, eye regions are either clearly valid or clearly invalid by the criteria mentioned above. Qualitatively approximately 1–2% of eye regions were difficult for the human annotator to classify. These cases require a judgement as to what is too much occlusion or too much motion blur. There is not a right or wrong answer here, but it is important to highlight the existence of such cases and their approximate frequency. The upper limit of the accuracy of any classification algorithm applied to this dataset will be limited by this frequency.

In the second stage of labeling, the locations of the pupil center and both corneal reflections were determined manually for each of the valid eye regions. As corneal reflections are small and well defined in the images they were labeled by the annotator with a single click. Single click estimation introduces quantization errors associated with the mapping of a continuously varying corneal reflection center location with an integer [x,y] pixel location. The labeling procedure will result in a quantization error of approximately 0.25 pixels in both the x and y direction, or an average error magnitude of approximately 0.35 pixels (assuming the probability density function of the quantization error is uniform). To label the pupil center the annotator first clicks on 10 points along the pupil–iris boundary. These 10 boundary points are used by the OpenCV [51] computer vision and image processing library function cvfitellipse2 to fit an ellipse to the pupil–iris boundary. The center of the fitted ellipse is recorded as the ground truth pupil center location for our dataset. The uncertainty in the pupil location estimates was determined empirically by selecting an eye window from ten different subjects and labeling each of them ten separate times. The average magnitude of the deviation from the mean of each pupil estimate was 0.18 pixels horizontally and 0.29 pixels vertically (or 0.34 pixels total magnitude). Increased uncertainty in the vertical direction is expected due to interference with the upper eyelid occluding part of the pupil. Overall however, the labeling of the center of the pupil–iris boundary has sub-pixel accuracy.

From these 4000 labeled eye regions, we created four distinct datasets, each with one of the following image crop sizes around a random location near the center of the labeled pupil center: 64 × 64, 96 × 96, 128 × 128, and 256 × 256 pixels. These data sets allowed us to evaluate the performance of the feature extractor networks with different crop sizes around the eye. Finally, we used data augmentation techniques to artificially increase the size of each dataset from 4000 eye regions to 40,000 eye regions by including ten crops at ten random locations near each labeled pupil center rather than only one. We split each dataset into separate training and validation sets which contain eye regions from 80 and 20 of all the subjects (100), respectively. Testing the networks was done by using data that were generated during the experimental testing of the eye-tracker (i.e., the data were not used for training), which will be discussed in Section 4.

### 3.2. Classifier Network Performance

The classifier is responsible for determining if an image of an eye region is valid (i.e., it contains two corneal reflections and a pupil). To evaluate and select the parameters for the classifier network, we performed the following experiment. First, we generated 256 hyperparameter configurations of the base architecture from the set shown in Table 1. Any configurations which required more than the available 11 GB of GPU memory to initialize and train (for a 256 × 256 image crop with a batch size of 32) were discarded and new configurations generated to replace them. The range of parameters in Table 1 were selected such that the inference networks generated would cover a wide range of computational complexities. The smallest possible network (one CNN layer with four 3 × 3 kernels, 4× downscale, and no hidden fully connected layers) can run on any modern smartphone at high number of frames per second (>100). The largest possible network (five CNN layers each with 128 9 × 9 kernels followed by hidden fully connected layers of 2048 neurons each) is much larger than the largest networks in Googles MobileNetV2 [52] family of image processing networks, which runs at between 3 and 30 ms [53] per inference depending on the device and accelerator used. As we aimed to achieve an estimation rate of at least 30 frames/s cumulatively across all our networks and for both eyes (at least theoretically if our device had modern accelerators) our exploration of the trade-offs between inference quality and inference cost for a smartphone-based eye-tracker occurred between these two extremes.

Then we trained each specific architecture independently with the four datasets discussed previously. Each network was trained with a batch size of 32 for a total of 20 epochs. To permit early stopping and minimize overtraining, at the end of each epoch the network was saved only if the absolute difference between the average training and validation errors for that epoch was smaller than after all previous epochs. Each network was evaluated based on two metrics; the classification accuracy on the validation set, and the computational cost of one forward inference though the network. We measured this cost in millions of floating-point operations (mflops), and this value was provided automatically by the neural network training library that we used Tensorflow version 1.12 [54].

**Figure 10:**
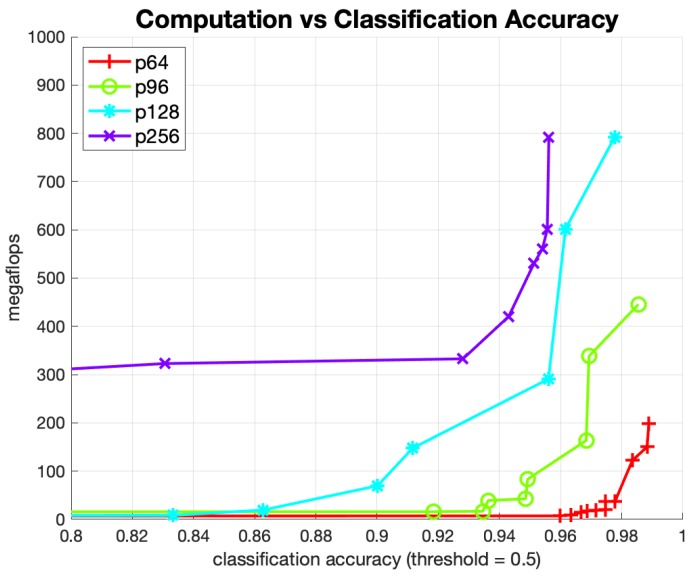
Pareto Classifier Networks: Performance vs. Cost vs. Crop Size.

Figure 10 shows the Pareto-optimal networks for this experiment, where each point in the figure is a specific instance of a trained network. The *x*-axis is the classification accuracy using a probability threshold of 0.5, thereby for an eye region to be classified as “valid” the output probability of the network for that class was over 50%. The four curves correspond to the size of the input image crop that was used to train and validate data for each curve. Observe that the bottom curve, corresponding to a 64 × 64 input image crop, is superior to all the other curves. The implication is that training on a smaller eye region crop produces similar or better classification accuracy with much less computational effort. As an example, to achieve a classification accuracy of 98% on the 128 × 128 pixel dataset required a network that used roughly 30 times more computation than to achieve the same accuracy on the 64 × 64 dataset.

As our manual labeling of the images requires a judgement as to when an image has too much pupil occlusion the final chosen configuration, which achieved accuracy of 98%, is near perfect given the slight subjectivity of the human-annotated classification labeling.

### 3.3. Feature Extractor Network Performance

We evaluated the pupil center and corneal reflections position estimation networks similarly to the classifier above. We selected 256 hyperparameter configurations for the base architecture randomly from the set of choices outlines in Table 1. Each network was trained with a batch size of 32 for a total of 20 epochs. To permit early stopping and minimize overtraining, at the end of each epoch the network was saved only if the absolute difference between the average training and validation errors for that epoch was smaller than after all previous epochs. Each network was evaluated based on the mean distance (measured in pixels of the original image) between the networks output estimate and the ground truth locations in the validation set. For brevity, this is shown primarily for the pupil-center estimation, although results are similar for the corneal reflections.

**Figure 11:**
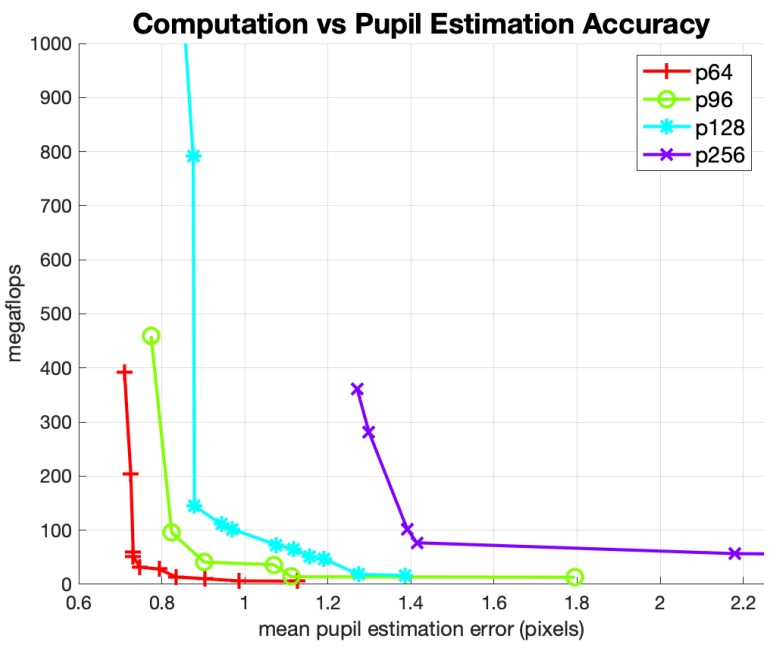
Pareto Pupil Regression Networks: Performance vs. Crop Size.

Figure 11 shows the Pareto-optimal curves for the suite of pupil estimation networks using the center-of-mass output layer described in Section 2.2. The *x*-axis is the mean estimation error (in pixels), and the *y*-axis is the number of mflops required to compute a single estimate. We reported the mean pupil estimation errors in this figure after eliminating estimates that had significant errors greater than 7.5 pixels. The removal of large errors is done to improve the comparison between networks trained on different image crop sizes. Otherwise, this type of analysis would favor smaller image crop sizes because gross errors in larger image crops are higher (in the absolute number of pixels) than gross errors in smaller image crops. Even with this correction, Figure 11 shows significant advantages for networks trained with smaller crop sizes. These advantages extend to both the performance-to-cost ratio and absolute estimation accuracy. This result led us to select the 64 × 64 image crop size for our pupil-center estimation network.

The improvement in both the computational load *and* the mean estimation error when training networks with tighter crop sizes is an interesting and counter-intuitive result. As a 64 × 64 crop is a direct subset of a 256 × 256 crop (of the same eye) in principle networks trained on the larger crop should be slower but get equivalent results. However, the 256 × 256 crop has extra information which is not relevant to the estimation of the pupil–iris boundary (the only feature relevant to estimating the pupil center) and thus the larger crop size presents an image with more nuisance parameters (i.e., lower signal-to-noise ratio). Training length, learning rate and optimizer explorations did not eliminate this effect. Training with 10–100 times more data might have an impact on this effect, but that effort is beyond the scope of this paper. In practice, when training small networks with small datasets it is important to limit the number of nuisance parameters.”

**Figure 12:**
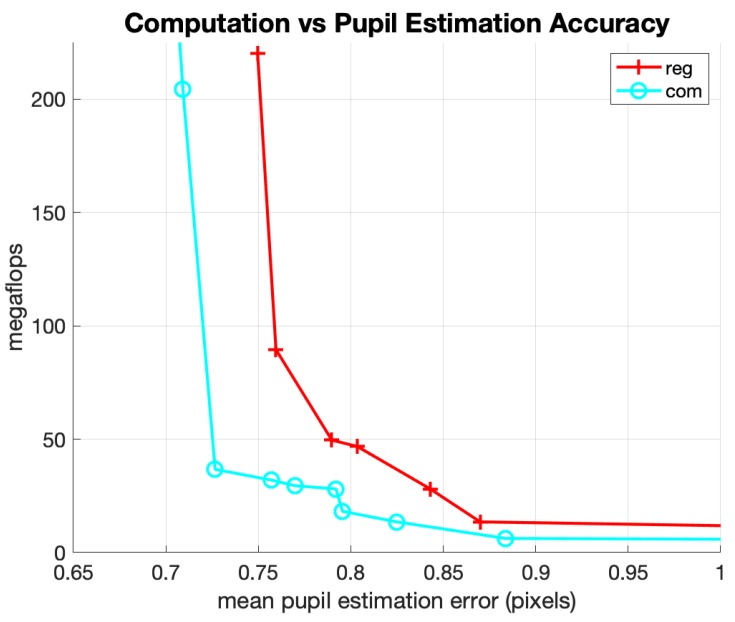
Pareto Pupil Regression Networks: 64 × 64 Crop w/wo Center of Mass.

The next experiment was to evaluate the performance of the pupil position estimator with and without the center-of-mass output layer. Figure 12 shows the Pareto-optimal curves for networks using simple regression and networks using the center-of-mass output layer. Across all 256 random hyperparameter configurations for the 64 × 64 cropped dataset, the average estimation error was improved by 14% by using the center-of-mass calculation. Near the best accuracy to cost ratios of the Pareto-optimal curves, this improvement was roughly 10%. Combining the improvements from both tighter image crops and the center-of-mass output layer, the net reduction in pupil estimation error is about 50% (at any given amount of mflops) when compared with a naive single CNN regression network trained on a 256 × 256 image crop.

Finally, we selected one specific configuration for each of the six networks in the feature extractor. Recall that this system consists of two small locator networks to quickly localize the pupil, a classifier to validate that the cropped image included a “valid” eye, and three accurate position estimators to estimate the pupil center and corneal reflections precisely. We selected these specific networks from points on the corresponding Pareto-optimal curves which represent the good trade-off between performance (error) and computational cost. Table 2 shows the image crop sizes, amount of down-scaling where applicable, average absolute error (in native pixels of the original unscaled image crop) between predictions and feature labels on our validation set, and computational cost of these networks (in megaflops).

For the course pupil and medium pupil networks (used to localize the 64 × 64 eye region crop) we explored down-scaling factors up to a maximum of eight rather than four because those networks required less spatial accuracy and needed to be as fast as possible. This localization step could have been done with a single 256 × 256 crop size network, but would have been more expensive to achieve the same average pupil estimation error. In our dataset the size of bounding boxes centered at the pupil center and containing both the entire pupil and both corneal reflections were as large as 58 pixels (for subjects with large pupils holding the phone close to their face). As a result, we would like most of the pupil localization estimates to have less than 6 pixels of error horizontally and vertically to avoid clipping part of the required eye features. This was more cost effective to achieve with a two-stage hierarchy.

There was significant flexibility in the selection of the hyperparameters that generated the best results. In most cases, we could have selected a different network on the Pareto curves that looked quite different but performed very similarly. For example, a network with similar performance might have had more CNN layers, but fewer kernels per layer, resulting in approximately the same overall computational requirements and accuracy. With that said, Table 3 shows the specific architecture parameters for the networks we selected for completeness. For each of the five networks Table 3 presents the number of kernels (k) and kernel width (w) for each CNN layer, the number of neurons in the hidden dense layer, and the dropout percentage between the hidden dense layer and the output layer. Pooling was enabled for all the networks we selected. The positive effect of pooling is not surprising for the classifier networks but was surprising for the feature estimation networks. It seems that the loss in spatial resolution from pooling CNN output feature maps was less critical than the gains from adding more kernels when normalized for total compute requirements. As a result, all selected networks had pooling enabled for every CNN layer. In the next section, we use the above networks to evaluate the end-to-end mobile eye-tracker.

## 4. Eye-Tracking Results and Comparisons

In this section, the performance of the hybrid eye-tracking system is evaluated and compared with three other mobile systems: Screen Glint [36], GazeCapture [35], and the previous version of our system [43]. We present these comparisons through a series of experiments. The 8 subjects who participated in both experiments did not contribute data to either the training or validation section of the dataset, which was described in Section 3.1.

Gaze-estimation models for stationary eye-tracking systems that are similar to the one that we used for our mobile eye-tracker have been extensively evaluated in the literature [29,41,55,56] with a variety of camera and light source configurations. These studies demonstrated that when robust eye features are provided to this 3D gaze-estimation model, high-quality gaze estimates can be produced in the presence of relative motion between the subject and the system. The purpose of the experiments in this section are to validate how well inexpensive CNNs can produce robust eye features in the presence of larger relative motion than was exhibited in these previous studies and when willing participants use a smartphone naturally in an indoor environment for the purpose to eye-tracking. As a result, the number of participants in this part of the study did not need to be large.

### 4.1. Eye-Tracker Performance at Different Distances

**Figure 13:**
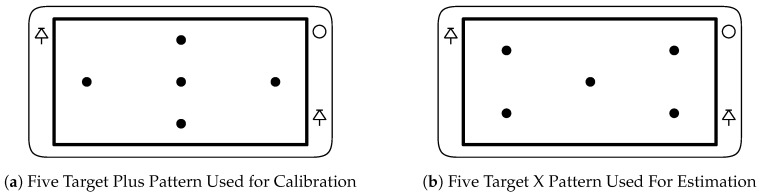
Sample Target Patterns Used for Calibration (**a**) or Estimation (**b**) (Adapted from [42]).

During the evaluation of the eye-tracker performance subjects were instructed to hold the smartphone in any position or orientation they felt comfortable. Then, the experimenter adjusted only the distance between the eye-tracker and their head to be approximately 30 cm at which point a calibration procedure was performed. During this calibration procedure five targets were shown on the display, one at a time, in the ‘plus’ pattern as shown in Figure 13a. As the subject gazed at each target the system collected 50 sets of eye features. The Levenberg Marquardt nonconvex optimization algorithm [57] was used to estimate the subject specific parameters which minimized the distance between the gaze estimates generated with those eye features and the corresponding target locations. These subject specific parameters were saved and used in the next part of the experiment.

Once the calibration parameters were estimated the experimenter adjusted distance between the eye-tracker and the subjects’ head to one of five approximately fixed distances; 20, 25, 30, 35, and 40 cm. Subjects’ were instructed to avoid large intentional movements, but that small natural head and device movement was both acceptable and expected. At each of the five distances each subject was asked to look at five target locations. The targets were displayed one at a time in the pattern shown in Figure 13b. For each target, the system collected 50 eye-gaze estimates using the calibration parameters generated in the previous step.

The average distance (in mm) between a target location and the mean location of 50 gaze estimates on each target were used as an estimate of a gaze bias. This value was converted into degrees using the approximately known distance between the subject and the device and then averaged across all 5 targets. Table 4 presents the average gaze-estimation bias, in degrees, at each distance and compares that to other mobile eye trackers. In that table, we refer to our previous work [43] as SmartEye and the current work as HybridTrack. GazeCapture did not present results at fixed distances, so for comparison, we only provided the average gaze bias. To more fairly compare this work with our previous work we reran SmartEye to produce gaze estimates on the same videos used in this work. As a result, the gaze bias results for SmartEye in Table 4 are slightly different than what was presented for a similar experiment in [43], but show the same trends.

It is clear from Table 4 that both the SmartEye and the HybridTrack have much lower gaze estimation bias than ScreenGlint or GazeCapture. The comparison between SmartEye and the present work also shows the improvement in robustness of the new mobile eye-tracker as it can maintain high gaze-estimation accuracy in the presence of significant distance variation. Please note that at the calibration distance of 30 cm, SmartEye performed very well (better than HybridTrack) with a gaze bias of only 0.57${}^{\circ }$ gaze. However, changing the distance by 10cm resulted in significant performance degradation (bias to as high as 1.90${}^{\circ }$). The new HybridTrack eye-tracker produced gaze-estimation bias results with much smaller variation (0.65${}^{\circ }$ to 0.81${}^{\circ }$) over the range of distances. An individual break down of the gaze performance for each of the 8 subjects tested in this work is shown in Table 5. This table also includes the eye color of each subject (light for blue hues, dark for brown hues). The contrast between the pupil and iris is worse for users with blue irises since their irises do not reflect as much IR as subjects with brown irises. This can lead to less robust feature extraction of the pupil–iris boundary and less accurate gaze estimates. The use of a CNN feature extractor trained on a wide range of iris colors mitigated this effect as the final gaze-estimation errors are similar for both groups. The sample size of only two light eye subjects would need to be increased however to come to any definitive conclusions.

### 4.2. Unsupervised Eye-Tracker Use

The second experiment was designed to evaluate the performance of the hybrid eye-tracker from the perspective of a software developer who wants to make use of the tracker in a broader application. When deploying an application that uses eye-tracking, the user can be given guidance on to how to hold and use the device, but its use would be in an unsupervised environment where the position and orientation of the device are unknown. Additionally, when the eye-tracking system indicates that a subject is looking at a specific target, the application developer would be interested in the certainty of such an observation.

**Figure 14:**
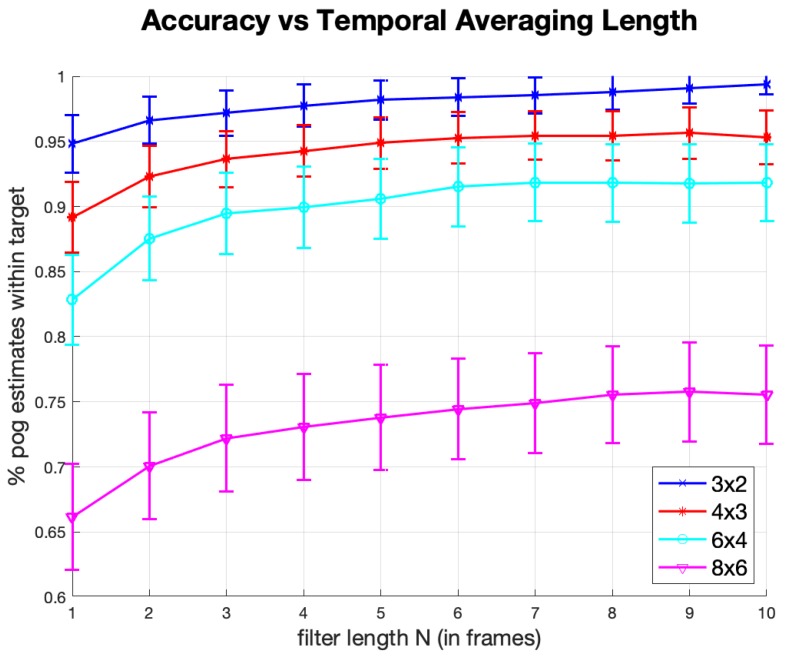
Target Identification Accuracy vs. Filter Length.

With this in mind, we designed the following experiment. We segmented the screen of the display into a grid of boxes that are approximately square target regions. Then, in a random order, each grid box (target) was highlighted, indicating to the subjects that they should direct their gaze to that region. Following a 500 ms delay, the system recorded 15 gaze estimates. We refer to this procedure as a grid test. One metric derived from this test is the percentage of gaze estimates that were inside the highlighted box. It is common, in eye-tracking, to also compute a single gaze estimate from a sequence of *N* consecutive estimates, to reduce RMS error through averaging. We show the results of averaging 2 to 10 estimates in Figure 14.

The specific experimental procedure for the grid test was as follows. Each one of eight subjects who participated in the study was asked to hold the device comfortably. Next, the subject performed a calibration procedure. The calibration parameters were stored and used in each subsequent session with that subject. Then, at three different times over the next five days, each subject was asked to perform the grid test at four different grid sizes; 3 × 2 (38.3 × 32.5 mm boxes), 4 × 3 (28.8 × 21.6 mm boxes), 6 × 4 (19.2 × 16.3 mm boxes), and 8 × 6 (14.4 × 10.8 mm boxes). After being given the device and the instructions, the test involved no experimenter supervision.

The calibration procedure of the infrared 3D gaze-estimation model used in this work estimates physiological geometries of a subject’s eye, which does not change over time. Therefore, we do not need to re-calibrate between each use of the eye-tracker after large relative movements between the subject and the device. This is a key property of our system when compared with other mobile eye trackers.

Figure 14 shows the percentage of gaze estimates that were within the correct target boxes as a function of the number of averaged estimates. Each point in Figure 14 is the average data for all 8 subjects for each experiment (i.e., a specific box size and number of averaged estimates). Take, for example, the 6 × 4 grid-size with number of averaged estimates of 4, Figure 14 indicates a 90% estimation accuracy. Given that we collected 15 gaze estimates for each target box, there are 11 gaze estimates for each target that use an average of 4 estimates. An accuracy of 90% indicates that of the 2112 total gaze estimates that use an average of 4 gaze estimates (11 estimates per target × 24 targets per subject × 8 subjects) about 1900 of them are located in the correct corresponding target boxes. Each point on the graph represents the mean performance of the eight participants. The error bars in Figure 14 show the standard error of the means

For the 3 × 2 grid (38.3 × 32.5 mm) Figure 14 indicates that 95–100% of all gaze estimates (depending on the number of gaze estimates that were averaged) were inside the highlighted boxes. The percentage drops with each increase in grid dimension and the corresponding decrease in box size: 89–96% for the 4 × 3 grid (28.8 × 21.6 mm boxes), 83–93% for the 6 × 4 grid (19.2 × 16.3 mm boxes) and 66–76% for the 8 × 6 grid (14.4 × 10.8 mm boxes). The performance difference between the 6 × 4 and 8 × 6 grid sizes indicates the limit of the effective performance of our system and testing smaller grid sizes would not be valuable.

**Figure 15:**
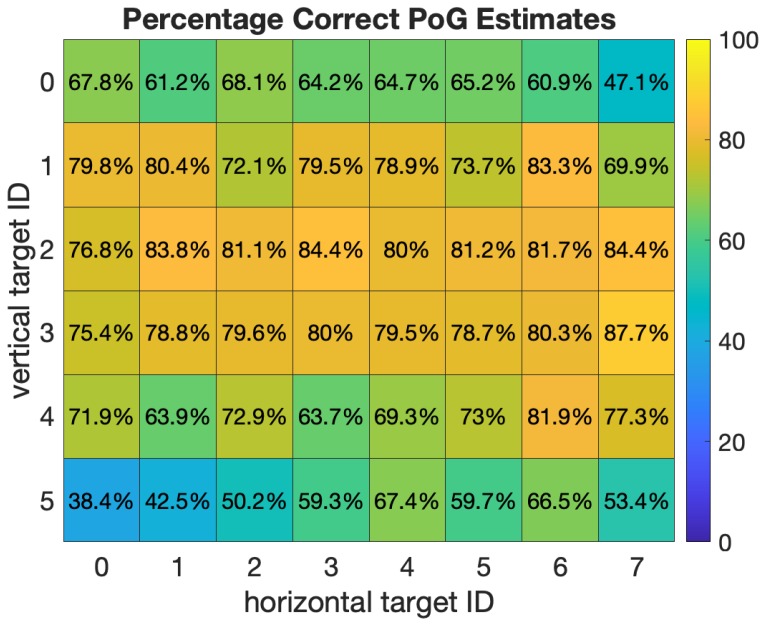
Spatial Distribution of 8x6 Grid Target Identification (14.4 × 10.8 mm boxes).

Figure 15 shows the results of the grid test on an 8 × 6 grid (14.4 × 10.8 mm boxes) by giving the percent correct estimations at each grid location. These results are across all 8 subjects with N (the number of gaze estimates that are averaged) set to 10. While the average across all boxes was 76%, one can observe significant variations in performance for specific locations on the screen. Gaze estimates in the central area of the display are more likely to be correct, with percentages in the high 70 s to low 80 s. Estimates closer to the corners of the display can be quite less, with the worst occurring at the bottom left corner box, with only 38% being correct within the box. We suggest that future authors also present this level of spatial gaze-estimation detail in their evaluation of mobile eye trackers, rather than a single average estimation bias.

We hypothesize that the principal explanation for this effect is associated with the physical locations of the LEDs on the prototype smartphone. The two LEDs on our prototype are near the top right and bottom left of the display, and these are the two regions with the worst performance. When a subject gazes near these regions one of the corneal reflections, being furthest from the pupil center, may not be created by the spherical region of the cornea. Our model [29] assumes that the corneal reflection is created by a spherical cornea, and so this can result in additional gaze bias. One solution to this problem on desktop systems is to have more LEDs (more than two) and to select the two corneal reflections closest to the pupil center when estimating the gaze. This solution might be less desirable on a smartphone due to power and space constraints.

## 5. Conclusions and Future Work

We have presented a new hybrid eye-tracking system for smartphone. The new system can achieve gaze-estimation bias of 0.72${}^{\circ }$ in realistic unsupervised scenarios across 8 subjects. This performance is achieved with a single calibration, operating over a realistic range of device positions (20–40 cm) and orientations. The hybrid eye-tracking system uses machine-learning algorithms to improve the robustness and accuracy of eye-feature estimation (the location of corneal reflections and the center of the pupil). The eye features are then used by a gaze-estimation model that is insensitive to relative motion between the subject and the smartphone. When handing the device to subjects with little to no instructions, the system could achieve better than 90% accuracy distinguishing between targets in a 6 × 4 grid (using simple averaging of five or more gaze estimates). Our hybrid eye-tracking system (which has the advantage of infrared illumination) achieves significantly better accuracy (more than 400% better) than the accuracy achieved by previous systems that use natural light and appearance-based methods to estimate gaze position. The improvement in performance comes from the hybrid approach: a machine-learning feature extraction step followed by a geometric 3D model. This improvement may enable mobile applications that require analysis of visual scanning behavior of subjects that view a limited number of items on a smartphone screen.

An infrared mobile eye-tracking system has several limitations, however, and its performance will be affected by operational parameters not explored in this work. To achieve the performance reported in this paper the user must be holding the device such that the head/eyes are in the frame captured by the front-facing camera of the handheld device and neither are occluded by clothing or accessories [28]. Infrared eye-tracking will naturally suffer from direct sunlight interference and is generally not suitable in bright outdoor environments when operated in direct sunlight. Operational parameters such as rapid changes in eye illumination due to rapid changes in the distance between the smartphone and the subject, shakiness resulting from use during transport, and reflections of the IR light from eye-glasses would all likely cause a reduction in performance. Solutions to these issues can be both use-case and hardware dependent. One possible general approach to help overcome these limitations however is to augment our infrared-illumination-based hybrid approach with gaze-estimation techniques that use natural light [35,36,58]. In a dual-model system, situations when infrared eye features are difficult to localize the less accurate methods that use natural light can be used to improve the range of mobile eye-tracking-based applications. Another limitation of our eye-tracking system is the need to have a smartphone with a front-facing infrared camera and at least two infrared LEDs. This configuration is currently available only on a limited number of high-end devices, and the access to this hardware is only available to the device manufacturers.

## Figures and Tables

**Table 1:** Base Architecture Hyperparameter Ranges.

Hyperparameter:	Choice Set
Input Down-Scaling Factor:	[1×, 2×, 4×]
Number of CNN Layers:	[1, 2, 3, 4, 5]
Number of Kernels in CNN Layer:	[4, 8, 16, 32, 64, 128]
Width of Kernels in CNN Layer:	[3, 4, 5, 6, 7, 8, 9]
Pooling at Each CNN Layer (2 × 2):	[enabled, disabled]
Number of Hidden Dense Layers:	[0, 1, 2]
Number of Neurons in Dense Layer:	[64, 128, 256, 512, 1024, 2048]
Dropout % of Dense Layers:	[0.3, 0.4, 0.5, 0.6, 0.7]

**Table 2:** Selected Networks: Size, Performance, and Cost.

Features Estimators	Crop Size (px)	Downscale	Error (native px)	Cost (mflops)
pupil coarse	256	8×	3.818	2.5
pupil medium	128	4×	1.602	2.51
pupil fine	64	n/a	0.746	31.95
cr-a	64	2×	0.515	11.64
cr-b	64	2×	0.529	12.55
			accuracy	
classifier	64	n/a	98%	20.07

**Table 3:** Selected Networks: Architectures.

Network	cnn l1		cnn l2		cnn l3		cnn l4		Dense		Cost (mflops)
	k	w	k	w	k	w	k	w	Neurons	Dropout	
p1	24	4 × 4	2	3 × 3	16	5 × 5	n/a	n/a	512	0.7	2.50
p2	24	4 × 4	2	3 × 3	16	5 × 5	n/a	n/a	1024	0.7	2.51
p3	8	4 × 4	32	6 × 6	8	3 × 3	32	6 × 6	1024	0.7	31.95
cr-a	64	5 × 5	8	3 × 3	64	5 × 5	n/a	n/a	1024	0.5	11.64
cr-b	16	4 × 4	48	3 × 3	48	6 × 6	n/a	n/a	1024	0.4	12.55

**Table 4:** Varied Eye-Tracker Depth.

System	20 cm	25 cm	30 cm	35 cm	40 cm	Average
GazeCapture	n/a	n/a	n/a	n/a	n/a	2.98${}^{\circ }$
ScreenGlint	n/a	3.38${}^{\circ }$	3.32${}^{\circ }$	n/a	2.32${}^{\circ }$	3.01${}^{\circ }$
SmartEye	1.90${}^{\circ }$	1.15${}^{\circ }$	0.57${}^{\circ }$	0.90${}^{\circ }$	0.98${}^{\circ }$	1.10${}^{\circ }$
HybridTrack	0.81${}^{\circ }$	0.70${}^{\circ }$	0.71${}^{\circ }$	0.73${}^{\circ }$	0.65${}^{\circ }$	0.72${}^{\circ }$

**Table 5:** HybridTrack Subject Specific Gaze Bias at Varied Eye-Tracker Depth.

Subject	Eye Color [Dark/Light]	20 cm	25 cm	30 cm	35 cm	40 cm	Average
01	dark	0.66${}^{\circ }$	0.58${}^{\circ }$	0.60${}^{\circ }$	0.59${}^{\circ }$	0.63${}^{\circ }$	0.61${}^{\circ }$
02	light	0.79${}^{\circ }$	0.81${}^{\circ }$	0.74${}^{\circ }$	0.66${}^{\circ }$	0.57${}^{\circ }$	0.71${}^{\circ }$
03	dark	0.85${}^{\circ }$	0.69${}^{\circ }$	0.62${}^{\circ }$	0.66${}^{\circ }$	0.51${}^{\circ }$	0.66${}^{\circ }$
04	dark	0.88${}^{\circ }$	0.75${}^{\circ }$	0.81${}^{\circ }$	0.83${}^{\circ }$	0.64${}^{\circ }$	0.78${}^{\circ }$
05	dark	0.73${}^{\circ }$	0.70${}^{\circ }$	0.72${}^{\circ }$	0.80${}^{\circ }$	0.62${}^{\circ }$	0.71${}^{\circ }$
06	light	0.79${}^{\circ }$	0.65${}^{\circ }$	0.62${}^{\circ }$	0.64${}^{\circ }$	0.72${}^{\circ }$	0.69${}^{\circ }$
07	dark	0.88${}^{\circ }$	0.74${}^{\circ }$	0.79${}^{\circ }$	0.83${}^{\circ }$	0.70${}^{\circ }$	0.79${}^{\circ }$
08	dark	0.95${}^{\circ }$	0.73${}^{\circ }$	0.81${}^{\circ }$	0.79${}^{\circ }$	0.78${}^{\circ }$	0.81${}^{\circ }$
combined	n/a	0.81${}^{\circ }$	0.70${}^{\circ }$	0.71${}^{\circ }$	0.73${}^{\circ }$	0.65${}^{\circ }$	0.72${}^{\circ }$
